# Supplementary material for: Competition for light color between marine Synechococcus strains with fixed and variable pigmentation
Source: Appl Environ Microbiol. 2025 Jul 24;91(8):e00087-25. doi: 10.1128/aem.00087-25 (PMC12366368; doi:10.1128/aem.00087-25)
Supplement: Supplemental figures — Figures S1 to S7. [file aem.00087-25-s0001.pdf]

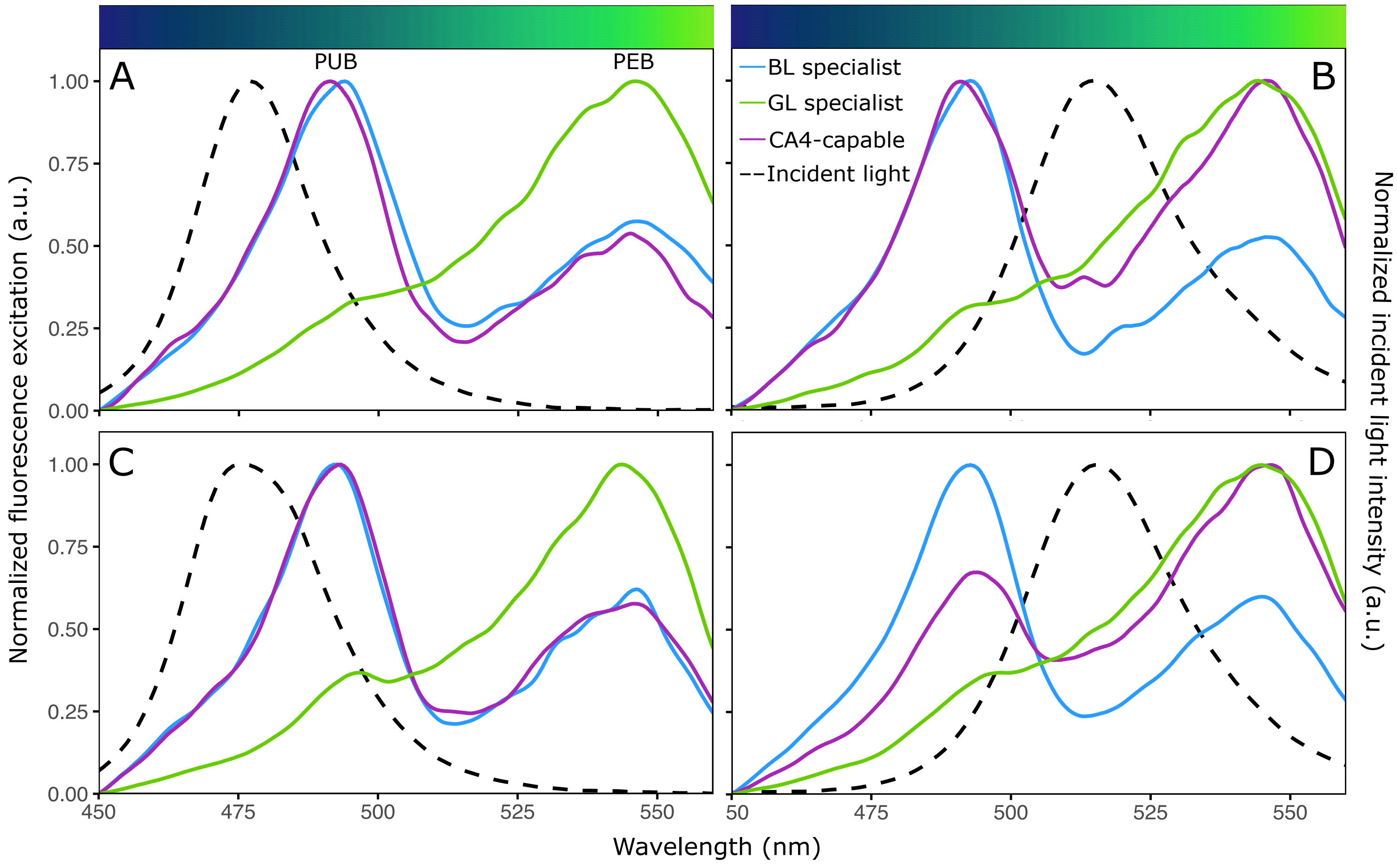

SUPPLEMENTARY FIGURE 1 | Representative excitation spectra of the three *Synechococcus* strains grown in the different light conditions used in this study. (A) Low blue light (LBL). (B) Low green light (LGL). (C) High blue light (HBL). (D) High green light (HGL). The LEDs spectra are represented by the black dashed line, while the excitation spectra of the green and blue specialists, as well as the CA4-capable strain, are illustrated in green, blue and purple solid lines, respectively.

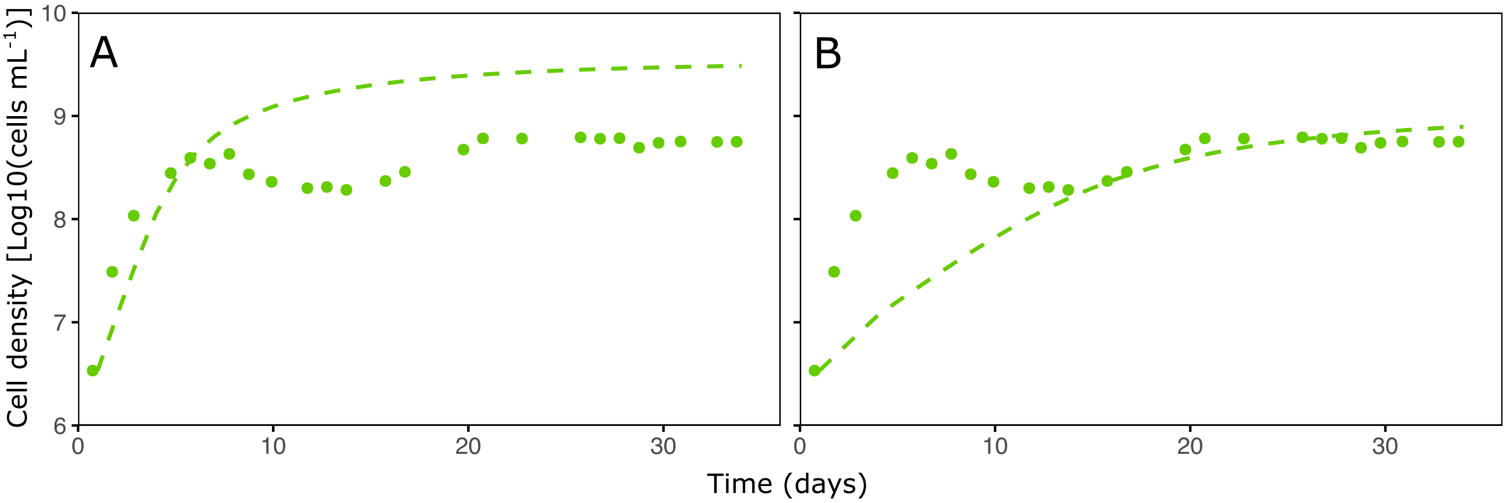

SUPPLEMENTARY FIGURE 2 | Example of model fitting to the data from the green light specialist in high green light (HGL). The model is able to capture either (A) the initial increase in population density but with an overestimation of the steady state population identity, or (B) the steady state population density but with an underestimation of the initial increase in population density. Points represent the data and lines represent the output of the model.

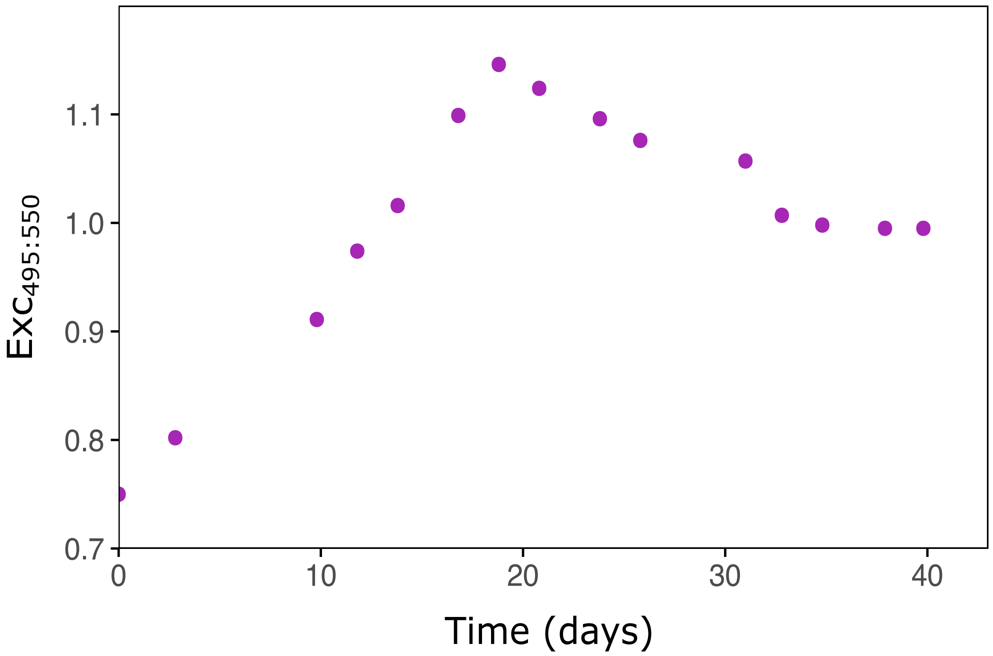

SUPPLEMENTARY FIGURE 3 | Time course variations of the $Exc_{495:550}$ fluorescence excitation ratio, a proxy of the whole cell PUB:PEB ratio, for the CA4-capable strain in the low green light (LGL) monoculture.

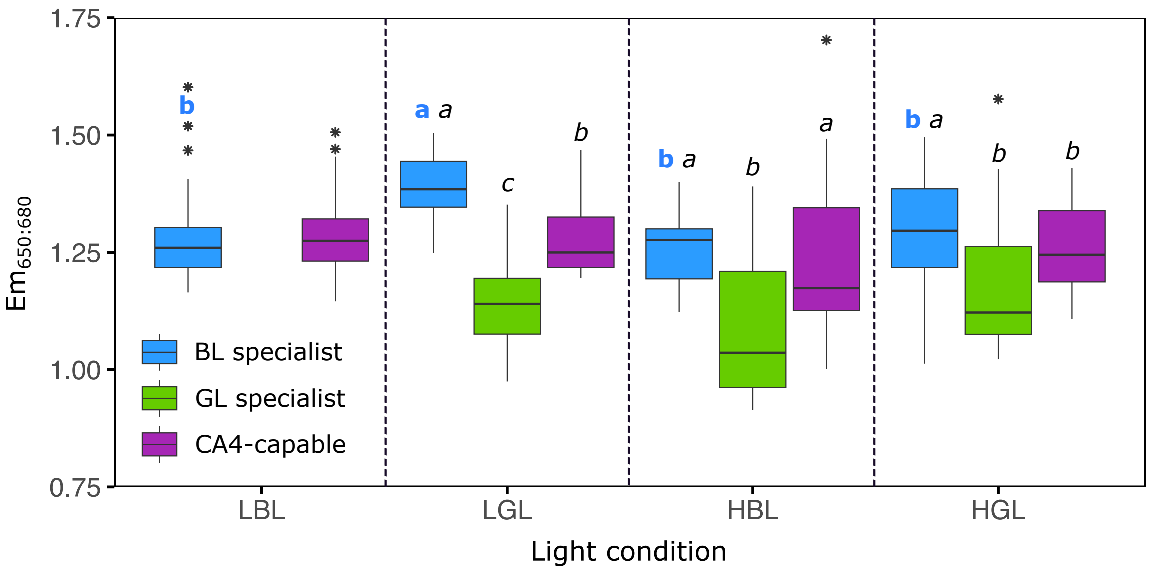

SUPPLEMENTARY FIGURE 4 | Mean Exc$_{650:680}$ fluorescence emission ratio, a proxy of the whole cell phycocyanin to terminal acceptor (PC:TA) ratio, for the three *Synechococcus* strains grown in monoculture in the different light conditions used in this study. Boxplots represent all measurements performed over the course of the experiments. The different letters above boxplots indicate statistical test results (one-way ANOVA followed by Tukey's test, or Kruskall-Wallis followed by Dunn's test). The first bold colored letter compares the values displayed by a given strain in the four different light conditions tested. The second italic black letter compares the three different strains in a given light condition.

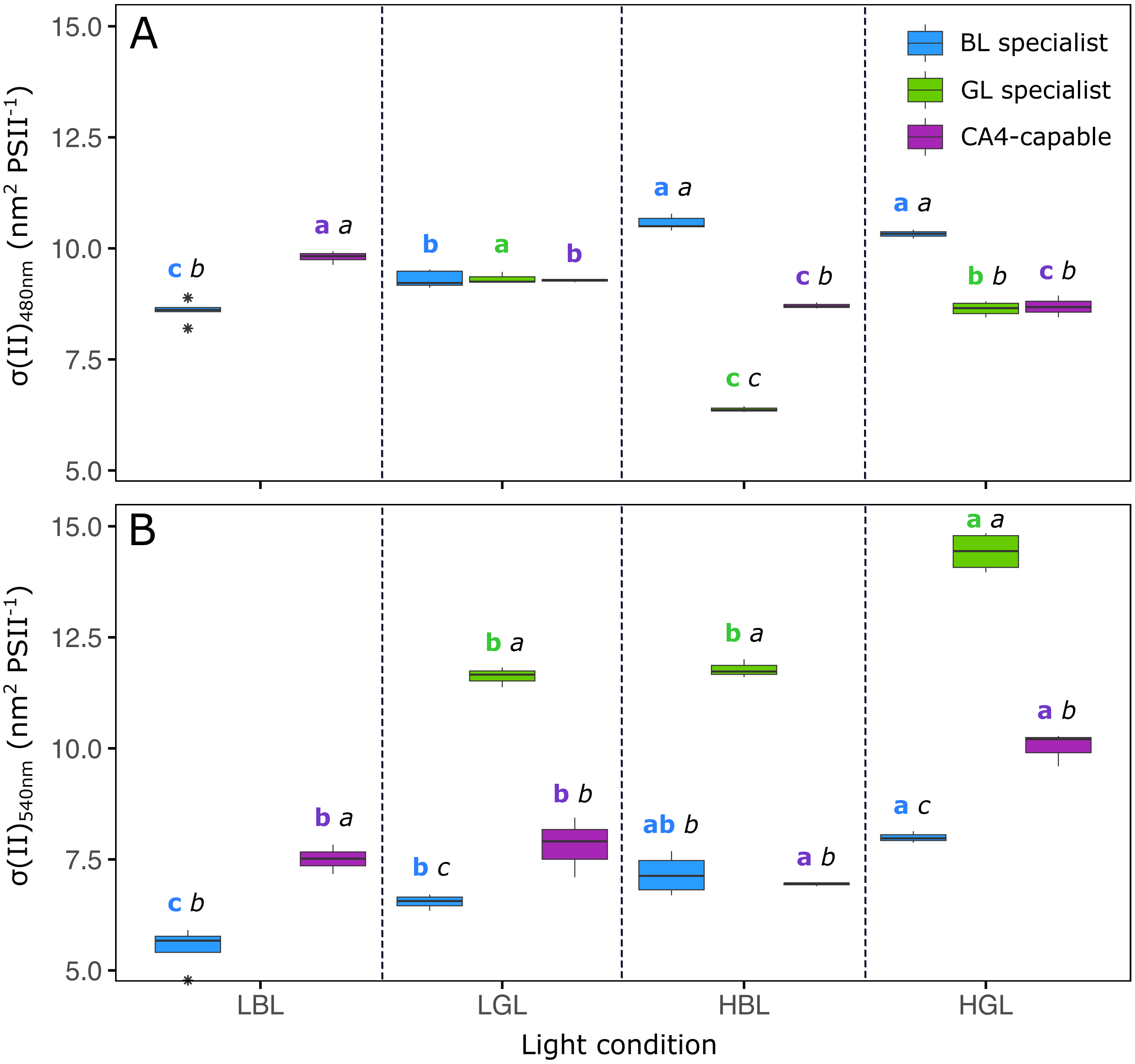

SUPPLEMENTARY FIGURE 5 | Photosystem II cross-section [σ(II)λ] for the three *Synechococcus* strains grown in monocultures in the different light conditions used in this study. (A) At 480 nm (cyan), the PAM excitation wavelength closest to PUB absorption peak (λ_{max} ≈ 495 nm). (B) At 540 nm (green), the PAM excitation wavelength closest to PEB absorption peak (λ_{max} ≈ 550 nm). Boxplots represent the ≥ 6 measurements performed during the growth phase and steady state for each strain and light condition. The different letters above boxplots indicate statistical test results for a specific wavelength (one-way ANOVA followed by Tukey's test, or Kruskal-Wallis followed by Dunn's test). The first bold colored letter compares the values displayed by a given strain in the four different light conditions tested. The second italic black letter compares the values exhibited by the three different strains in a given light condition.

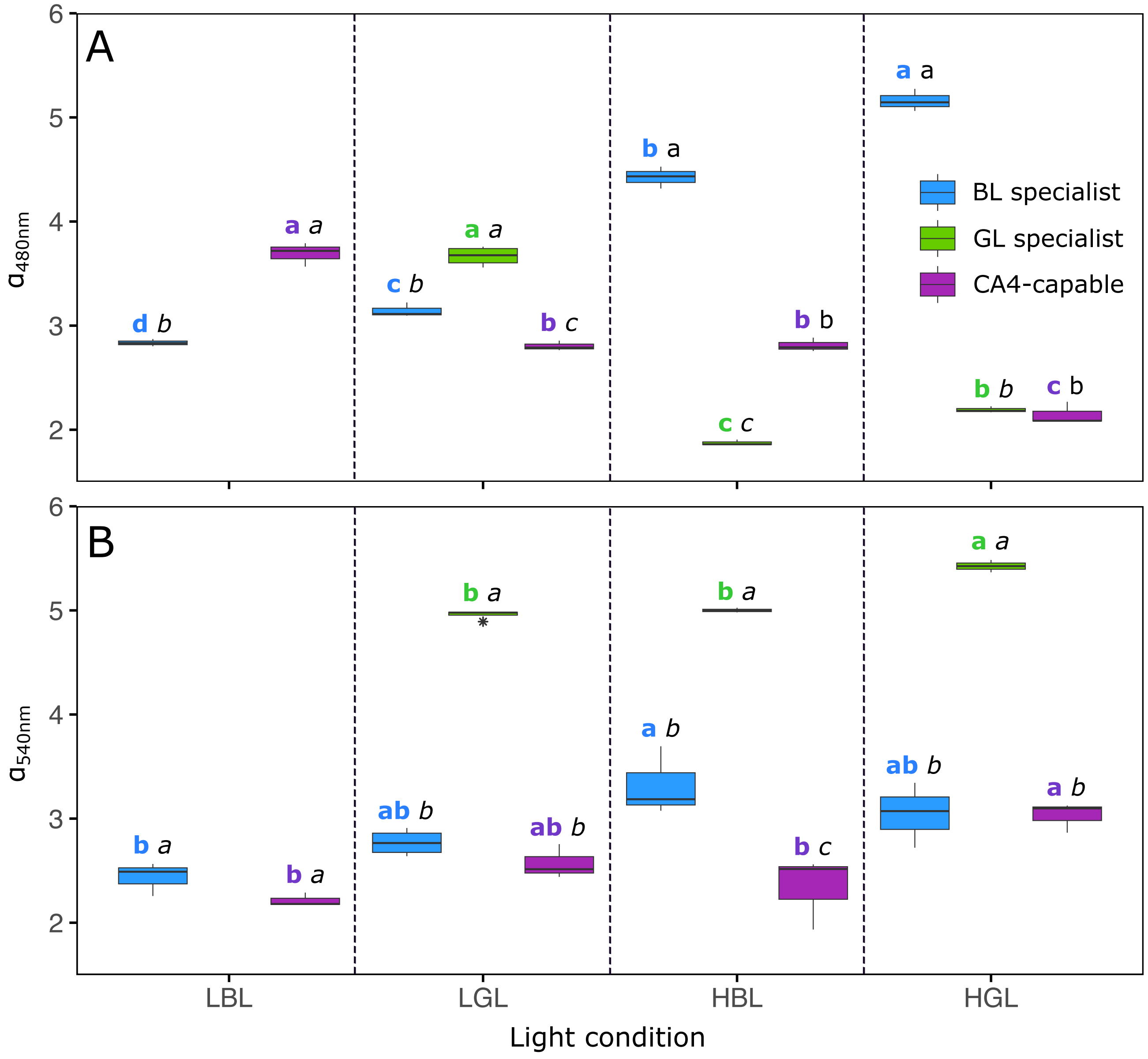

SUPPLEMENTARY FIGURE 6 | Same as Suppl. Fig. 5 but for photosystem II efficiency under non saturating light (α).

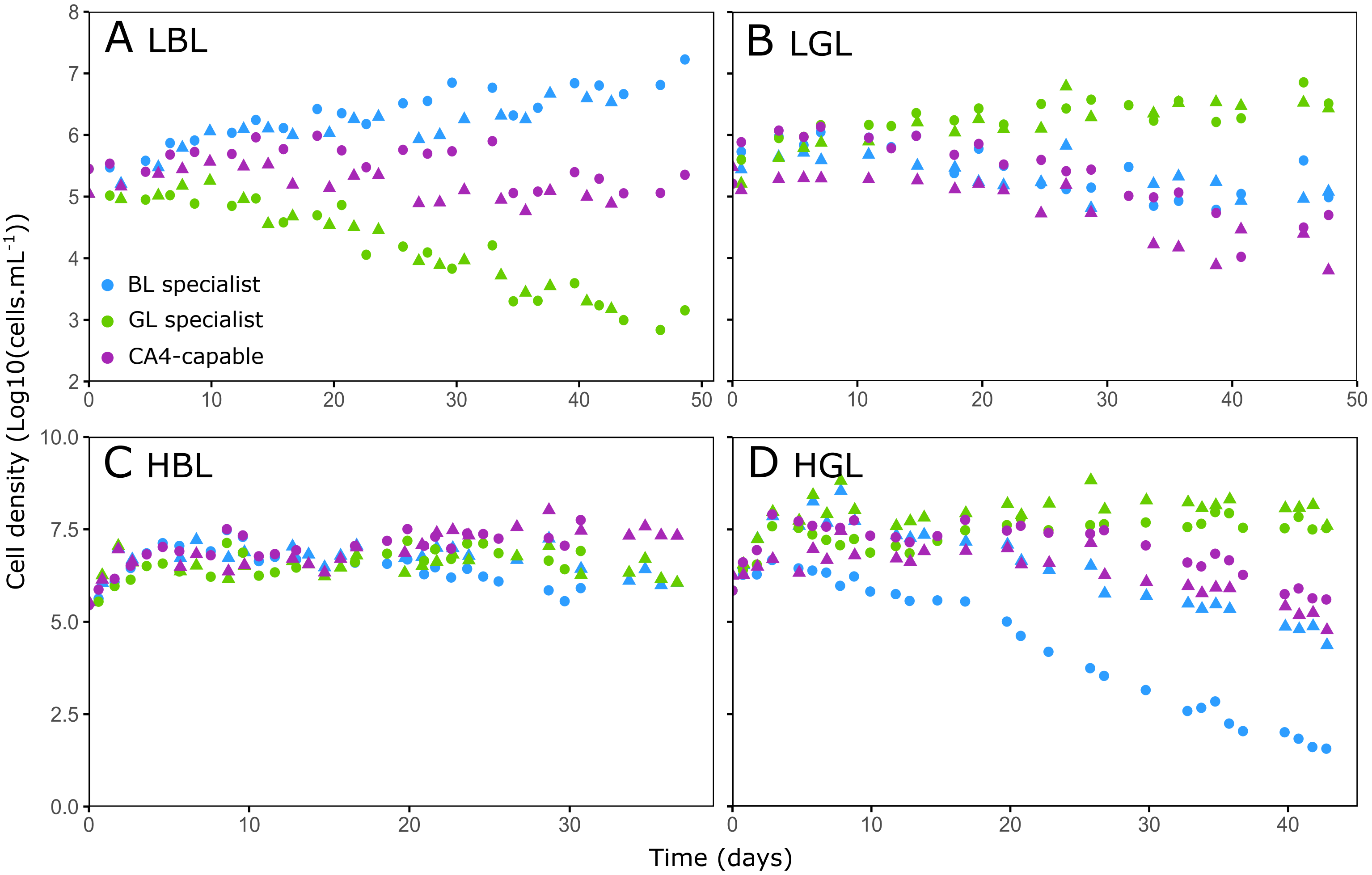

SUPPLEMENTARY FIGURE 7 | Time course changes in co-cultures of the cell density of the three marine *Synechococcus* strains in the four tested light conditions, as determined by a combination of qPCR and flow cytometry approaches. Note that this figure is similar to Figure 5 but the latter shows relative cell densities. (A) Low blue light (LBL). (B) Low green light (LGL). (C) High blue light (HBL). (D) High green light (HGL). Shapes indicate different replicates (circles for replicate A and triangles for replicate B).
